# Electrodeposition of Gold to Conformally Fill High Aspect Ratio Nanometric Silicon Grating Trenches: A Comparison of Pulsed and Direct Current Protocols

**DOI:** 10.4236/jsemat.2015.54022

**Published:** 2015-10-16

**Authors:** Sami A. Znati, Nicholas Chedid, Houxun Miao, Lei Chen, Eric E. Bennett, Han Wen

**Affiliations:** 1 Imaging Physics Laboratory, Biochemistry and Biophysics Center, National Heart, Lung and Blood Institute, National Institutes of Health, Bethesda, MD 20892; 2 Center for Nanoscale Science and Technology, National Institute of Standards and Technology, Gaithersburg, MD 20899

**Keywords:** Pulsed Electroplating, gold electroplating, high aspect ratio trenches, gold electrodepostion, direct current electrodeposition, pulsed vs. direct current electroplating, atomic layer deposition, platinum seedlayer, silicon trench gratings, trench filling, grating filling, ALD adhesive layer

## Abstract

Filling high-aspect-ratio trenches with gold is a frequent requirement in the fabrication of x-ray optics as well as micro-electronic components and other fabrication processes. Conformal electrodeposition of gold in sub-micron-width silicon trenches with an aspect ratio greater than 35 over a grating area of several square centimeters is challenging and has not been described in the literature previously. A comparison of pulsed plating and constant current plating led to a gold electroplating protocol that reliably filled trenches for such structures.

## 1. Introduction

The gold electrodeposition process has applications in numerous industries worldwide. For x-ray optics, especially in the hard x-ray regime, gold is a widely used absorbing and phase shifting material thanks to a high density and high refractive index (1). Conformal gold electrodeposition has found routine application in the filling of high-aspect-ratio trenches and the fabrication of x-ray absorption and phase gratings (2,3,4) and zone plates (5,6,7). To fill nanometer-scale trenches, this method of electrodeposition is only appropriate if a conductive seed layer is pre-coated over the substrate prior to the filling of the trenches. Conformal electroplating of silicon substrates was recently demonstrated for 100 nm wide trenches of 30 aspect ratio for macroscopic imaging applications (6). Electrodeposition of gold in the high aspect ratio, nanometric scale trenches requires a specific current flow across the surface to facilitate electrodeposition without damaging the grating structures or prematurely sealing the trenches. Here, the gold electroplating procedure and process parameters that led to conformal deposition of gold to coat high aspect ratio silicon trenches is described along with the steps for the fabrication of a substrate suitable for deposition.

### 1.1. Electrochemical Reaction

For the electrodeposition of gold films with a potential-driven electrolytic cell, reduction at the cathode leads to gold deposition. Reduction is achieved via electron transfer from the polarized substrate surface to dissolved Au(CN)_2_^−^ ionic complexes, splitting the complex and coating the substrate surface with solid gold. This electrolytic reaction occurs at the interface of solution and substrate surface. At this interface, the CN^−^ and Au(I) reactive species maintain the following equilibrium (8,9):

(1) $${\text{Au}}^{+}(\text{aq})+2{\text{CN}}^{-}(\text{aq})\to \text{Au}{{\left(\text{CN}\right)}_{2}}^{-}(\text{aq})$$

The reduction occurs at the cathode (platinum coated silicon substrate) as follows (9):

(2) $$\text{Au}{{\left(\text{CN}\right)}_{2}}^{-}(\text{aq})+e^{-}\to \text{Au}(s)+2{\text{CN}}^{-}(\text{aq})\;E{}^{\circ}=-0.37V_{\text{SCE}}$$

The electrodeposition is accomplished with the application of either a constant Direct Current (DC) or a pulsed direct current (PC) (10–12). For direct current, the power supply generates a constant current across the electrolytic cell, whereas for pulsed direct current the current is applied intermittently with pre-determined duty cycle ratio and length. The conformal electroplating of high aspect ratio trenches with both direct current and pulsed current electroplating protocols was evaluated experimentally and the two protocols were compared to determine the protocol and parameters most suited for filling nanometric scale, high aspect ratio silicon trenches with electroplated gold. The quality of the gold film was evaluated with scanning electron microscopy (SEM) and, combined with experimental results from x-ray studies, the best electroplating protocol and process parameters were determined from these results and replicated to fabricate x-ray phase contrast gratings.

## 2. Materials and Methods

### 2.1. Silicon substrate grating preparation

Silicon gratings of 200 nm and 400 nm periods were patterned with nanoimprint lithography and cryogenic reactive ion etched (RIE) to ~ 2 – 4 µm (13). A platinum seed-layer was atomic layer deposited (ALD) with a trimethyl(methylcyclopentadienyl)-platinum(IV) (MeCpPtMe_3_) and O_2_ recipe (10) to a thickness of ~10–30 nm over a 5 nm Al_2_O_3_ ALD adhesion layer with trimethyl aluminum (TMA) and H_2_O recipe.

**Figure 1:**
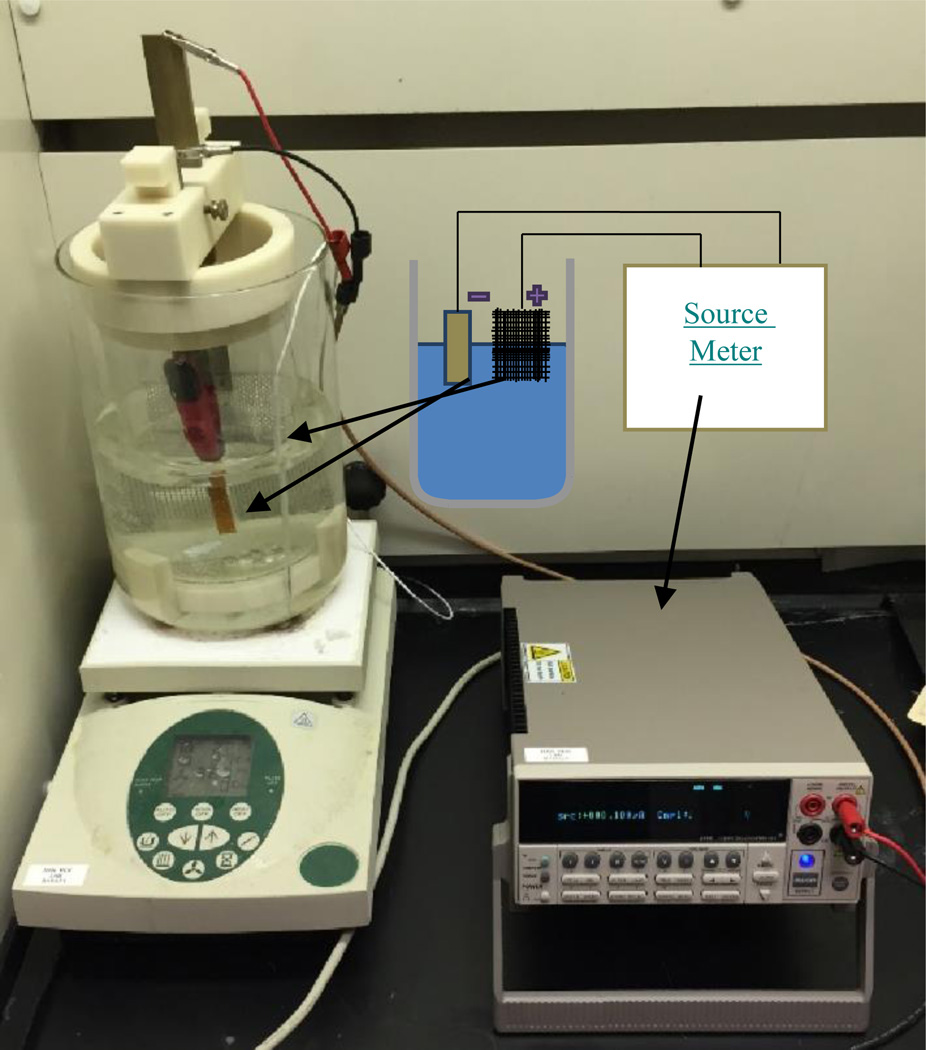
Electrodeposition Setup The electroplating setup with reactor (left) and sourcemeter (right).

After seed layer deposition, the substrate was cleaned thoroughly with ethyl alcohol, sulfuric acid and deionized water. The substrate was submerged with trenches oriented vertically into a cyanide based acidic (pH 6) gold electroplating solution at 323 K along with inert platinum-steel mesh anode and a magnetic stirbar. A cathode wire was clamped to the platinum surface of the substrate above the solution and connected to an 1100V source meter along with the counter anode. Figure 1 details the electrodeposition setup.

### 2.2. Electrodeposition Process

Both the direct current and pulsed current potentials were supplied long enough to completely fill the trenches. For the DC protocol, a constant current density of 0.04 mA/cm^2^ was applied to the reactor for 120 minutes. For the PC protocol, the source meter was set to supply a square wave current at various duty cycles for 100 minutes. The optimal pulse duty cycle and current density for this process was experimentally determined and a 50% duty cycle ratio (10 ms on, 10 ms off) with 0.08 mA/cm^2^ peak current density and 0.04 mA/cm^2^ average current density produced the optimal results for this process.

## 3. Results

### 3.1. Plating uniformity with respect to the depth of the trench

**Figure 2:**
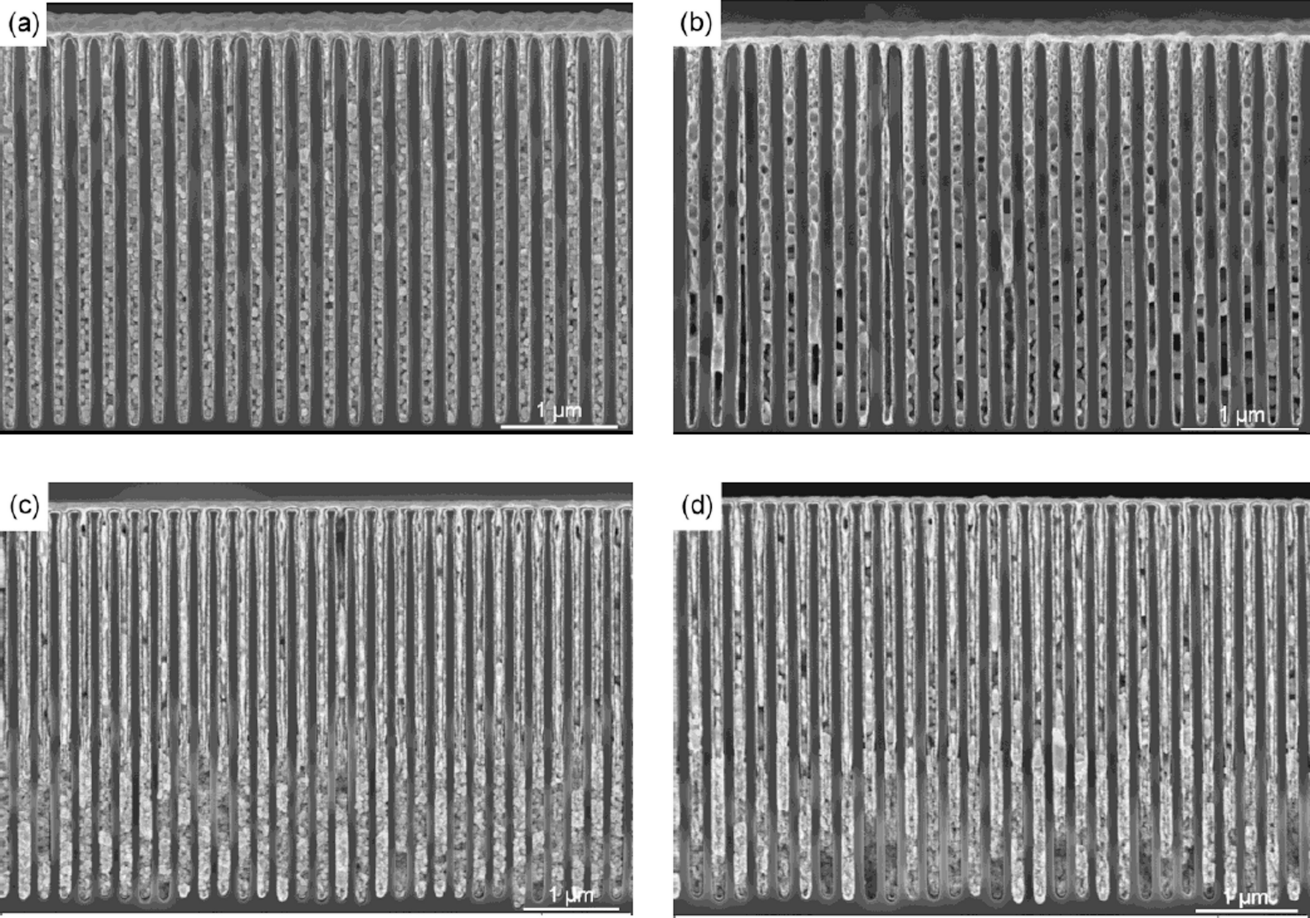
Direct Current and Pulsed Electroplating (a) and (b) are cross-sectional SEM images of a sample after DC plating, at a location near the surface of the plating solution and at the deep end of the sample, respectively. (c) and (d) are the same views of a pulse-plated sample. The DC plating exhibited considerable deposition rate variation with respect to the depth of the sample. This issue was addressed by the application of pulsed plating.

The DC protocol produced a gold deposition that varied along the length of the substrate, indicating the deposition rate increases with the solution depth for the DC protocol (Fig. 2a and 2b). Such gold density variation would lead to non-uniform x-ray refraction and poor performance of the phase grating. The PC protocol, however, displayed no rate variation with respect to solution depth (Fig. 2c and 2d) and the gold deposition formed comparatively more densely packed gold granules with improved gold trench filling.

### 3.2. Duty cycle pulse ratio and cycle length

Several duty cycle ratios and cycle lengths for the pulsed protocol were tested. The lowest pulsed duty cycle ratios produced more bulk crystals than the larger duty cycle ratios, however, the spacing and uniformity of the deposition was poorer. Longer pulsed cycles exhibited more variability from point to point along the substrate surface and fewer single crystal granules. The 50% duty cycle ratio with 20 ms cycle length produced the optimum deposited gold film after examination under Scanning Electron Microscope.

### 3.3. Effects of seed-layer morphology

**Figure 3:**
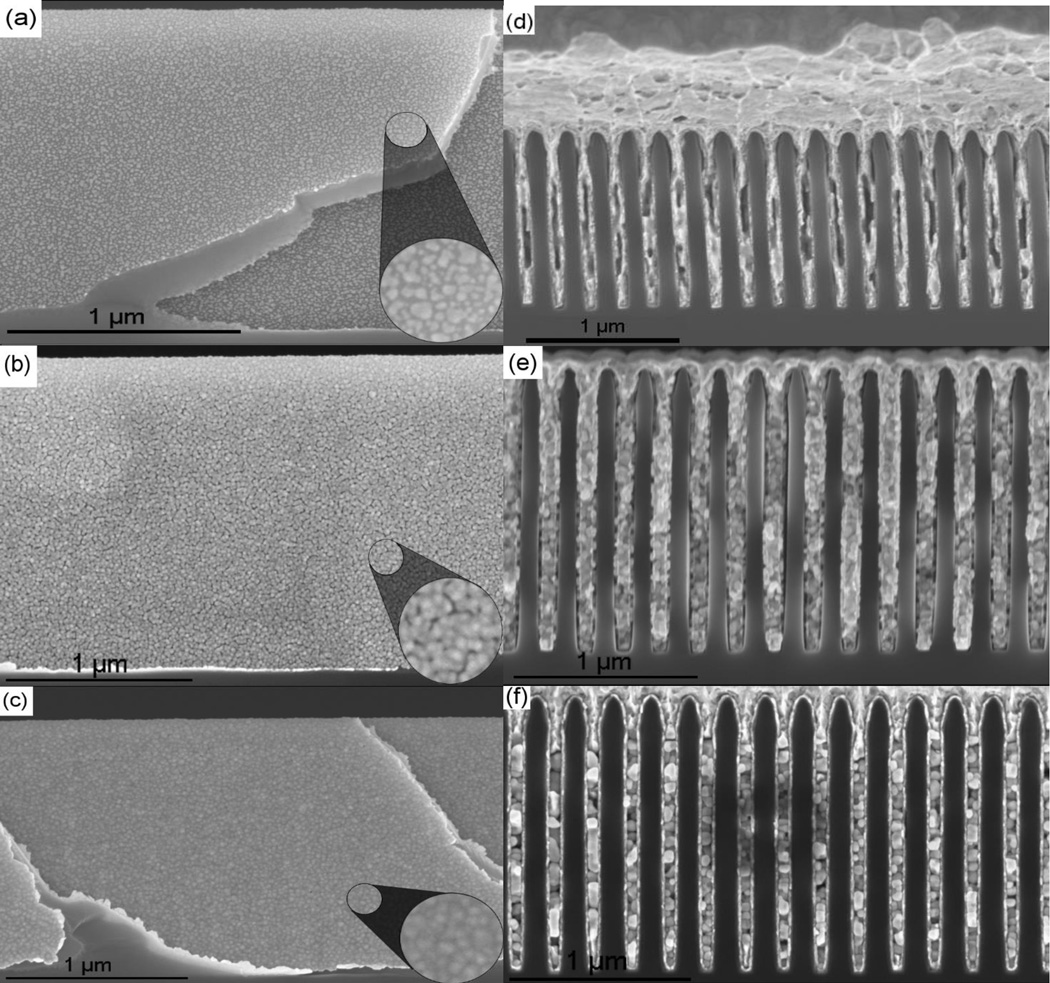
Direct Current Electroplating Side view of cleaved sidewall platinum seedlayer after (a) 200 loops, (b) 300 loops and (c) 400 loops of atomic layer deposition, and cross-sectional SEMs of the subsequently gold-filled 400 nm period trenches, with (d) ALD platinum deposition of 200 loops, (e) 300 loops and (f) 400 loops. The 3x magnified insets in (a) to (c) illustrate the progression from isolated platinum islands to contiguous seedlayer and the corresponding cross sectional SEMs (d) to (f) show the similarities between the seedlayer and the resulting gold deposition.

The gold electrodeposition process requires a conductive, electrically continuous surface to facilitate deposition. As previously stated, to produce a surface conducive to electroplating, the substrate was coated with a conductive material to serve as the seed-layer for the electrodeposition. Platinum was chosen as the seed-layer material because of high electrical conductivity and similar physical, chemical and x-ray properties to gold. During ALD of the seed-layer, disjointed platinum islands were formed and later coalesced to form a fused platinum surface. The platinum islands enlarged with each successive cycle until the entire surface of the substrate was covered with a uniform platinum seed-layer (Fig. 3a–c).

After 200 loops of platinum ALD, the seed layer primarily consisted of isolated platinum islands (Fig. 3a) and was too sparse for uniform gold deposition. Substrate coated with 200 loops of ALD platinum formed a disjointed seed-layer that led to partial gold trench filling with large pockets of empty space along the central trench line (Fig 3d). After 300 loops of platinum deposition, the platinum seed-layer completely covered the substrate surface (Fig. 3b). The resulting gold deposition was dense with large, tightly packed crystals (Fig. 3e). 400 loops of ALD produced a more uniform and less granular seed-layer (Fig. 3c) and electrodeposition facilitated by this seed-layer formed a densely packed gold deposition (Fig. 3f) with consistent formation of large bulk gold crystals and less deposition variation from trench to trench.

### 3.4. Electroplating without adhesion layer

**Figure 4:**
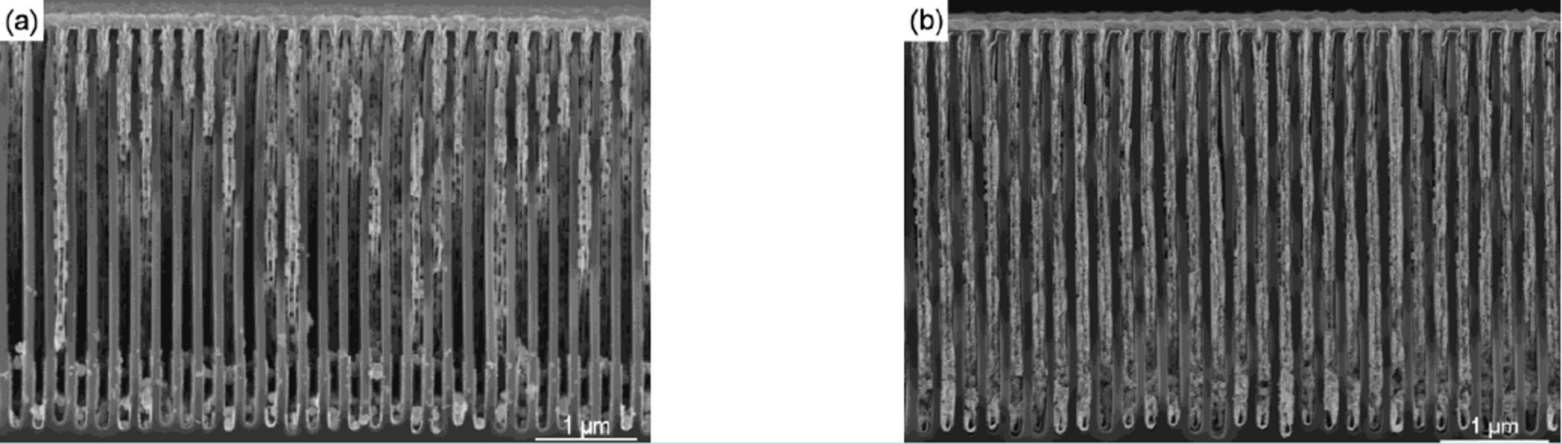
Electroplating without Adhesive Layer The result of (a) DC and (b) PC electrodeposition of gold following ALD of the platinum seed layer without prior Al_2_O_3_ adhesion layer. During the DC plating the seedlayer detached from the silicon surface (a), and led to incomplete filling of the trenches. The pulsed plating deposition (b) lacked the large segments of missing seed layer and gold deposition, suggesting the pulsed protocol deposited seed crystals that placed the interface under less stress and maintained the integrity of the ALD seed layer.

To further understand the difference between DC and PC plating, the DC protocol and PC protocol were tested with substrate fabricated without AlO_2_ adhesive layer and the platinum seed layer directly deposited onto the silicon surface. DC plating (Fig. 4a) led to substantial detachment of the seed layer from the substrate and partial filling of the trenches, while PC plating (Fig. 4b) caused minimal detachment and near complete filling of the trenches, consistent with the previous description of pulsed gold electroplating (14).

## 4. Conclusion and Discussion

Previous studies (15–17) have suggested that electroplating was a viable method to fill high aspect ratio trenches with gold. A comparison of pulsed and constant current electroplating of high aspect-ratio trenches described here and elsewhere (16,17) suggest the pulsed protocol produced more uniform and less porous deposition than the DC protocol. The pulsed plating deposition forms closer nucleation sites (Fig 2) and the proximity of the seed crystals leads to more uniform and regular gold film deposition.

The density of the gold nuclei formed during the electroplating process was previously shown (9) to increase with the magnitude of the applied potential. This would suggest the reduced electrical potential during the pulsed protocol relaxation period will produce seed crystals with lower density than those formed during direct current electroplating. The results obtained with this study suggest the opposite, as the pulsed protocol deposition is denser than that of the direct current: the lack of diffusion into the trenches is responsible for the lower gold density. The negative electrical potential of the substrate and the resultant precipitation of Au(s) reduces the ionic concentration of the interface solution and raises the energy required to complete the deposition reaction (9). The relaxation period of the pulsed protocol reduces the negative electrical potential at the surface and consequently permits the diffusion of Au^+^ from the bulk solution to the trench where the negative electrical potential applied to the substrate drives the reduction and consequent deposition of gold.

After each applied pulse, the interface Au^+^ concentration starts to return to bulk concentration and subsequent pulses initiate new sites for nucleation. This process will lower the energy required for deposition and keep the ionic concentration high enough to maintain Au reduction and deposition at the substrate surface.

The formation of seed crystal nuclei will place the substrate under stress and may affect or even detach the ALD seed-layer under certain circumstances. The effect of stress from seed crystal formation was shown to worsen without adhesion of the seed layer to the substrate, and the platinum seed-layer detached during direct current gold deposition without adhesion layer (Fig. 4a). The large crystalline structures formed during the DC plating protocol suggest the DC protocol forms less evenly spaced crystal nuclei than the pulsed protocol. Seed-layer detachment was less of a concern with the uniformly spaced nuclei of the pulsed protocol (Fig 4b) and suggest the spacing of the pulsed protocol crystal nuclei will lead to less force against the seed layer.

Even when the conformal electroplating was conducted with the pulsed protocol electrodeposition, the gold film deposited possesses a granular structure, especially with a film only twice as thick as the largest crystal formed. The electrodeposition along each side of the high aspect ratio trench draws gold from a shared reservoir of electroplating solution saturating the trench space. The simultaneous electrodeposition forces each side to compete for access to ionic species from the electroplating solution. Combined with the lack of ionic replenishment inside the trench during the last stages of electrodeposition, the concurrent electrodeposition at each side and the nanometric scale of the gold layers leads to the formation of pockets during trench closing. Despite such apparent defects under high magnification, the pulsed current gold deposition was formed evenly and without any large scale variation. The trench filling for the pulsed current deposited film was measured as equivalent to 87% bulk gold filling of the trenches by x-ray diffraction (13).

These fabricated trench gratings serve as the key component for phase-sensitive imaging techniques with phase interferometers (18). The conformal deposition of the gold film to fill 100–200 nm wide high aspect ratio silicon trenches is largely a result of the ALD platinum seed-layer and the application of a 50% duty cycle pulsed electroplating protocol to coat the trenches with gold.
